# Supplementary material for: Computational assessment of the relationship between metabolism and histone methylation in cancer cells
Source: PLoS One. 2026 Feb 13;21(2):e0340968. doi: 10.1371/journal.pone.0340968 (PMC12904441; doi:10.1371/journal.pone.0340968)
Supplement: S1 Table — This table summarizes the core properties of each dataset used in the study, including metabolome, transcriptome, chromatin profiling, and the integrated multi-omics dataset (Combined). For each data type, the initial number of samples, total number of features prior to quality control, and the number and nature of retained features following filtering are reported. The feature “nature” column specifies whether the retained variables include metabolites, gene expression values, chromatin accessibility/epigenetic marks, or concatenated features from multi-layer integration. This table provides a comparative overview of data dimensionality reduction and the biological meaning of remaining features across modalities. (DOCX) [file pone.0340968.s001.docx]

**S Table 1. Overview of data modalities, sample size, and feature characteristics before and after filtering.**

| Data type | Initial sample size | Number of features | Nature of features |
| --- | --- | --- | --- |
| Metabolome | 928 | 227 | Metabolites |
| Transcriptome | 1019 | 1928 | Transcripts |
| Combined | 870 | 2155 | Metabolites and transcripts |
| Chromatin profile | 897 | 26 | Histone methylation marks |
