# Supplementary material for: Computational assessment of the relationship between metabolism and histone methylation in cancer cells
Source: PLoS One. 2026 Feb 13;21(2):e0340968. doi: 10.1371/journal.pone.0340968 (PMC12904441; doi:10.1371/journal.pone.0340968)
Supplement: S2 Table — This table reports the canonical variates (CVs) that show statistically significant associations between metabolomic profiles and histone methylation features. For each significant canonical variate, the table includes the canonical correlation coefficient (Corr), F-statistic (F), numerator degrees of freedom (num df), denominator degrees of freedom (den df), and the associated p-value (Pr > F). These values indicate the strength and significance of the multivariate correlation structure between the two molecular layers. (DOCX) [file pone.0340968.s002.docx]

**S Table 2. Significant canonical variates from metabolome–histone methylation canonical correlation analysis.**

|  | Corr | F | Num df | Den df | Pr (>F) |  |
| --- | --- | --- | --- | --- | --- | --- |
| CV1 | 0.7123 | 1.28 | 5850 | 16783.8 | 2.20E-16 | *** |
| CV2 | 0.7026 | 1.22 | 5600 | 16159.2 | 2.20E-16 | *** |
| CV3 | 0.6826 | 1.17 | 5352 | 15532.8 | 3.78E-14 | *** |
| CV4 | 0.6523 | 1.13 | 5106 | 14904.4 | 1.28E-08 | *** |
| CV5 | 0.637 | 1.09 | 4862 | 14274.3 | 2.97E-05 | *** |
| CV6 | 0.6157 | 1.06 | 4620 | 13642.3 | 0.004543 | ** |
| CV7 | 0.6082 | 1.03 | 4380 | 13998.5 | 0.076742 | . |

Significant Codes: 0 ‘***’ 0.001 ‘**’ 0.01 ‘*’ 0.05 ‘.’
